# Different cation-protonation patterns in mol­ecular salts of unsymmetrical dimethyhydrazine: C_2_H_9_N_2_·Br and C_2_H_9_N_2_·H_2_PO_3_

**DOI:** 10.1107/S2056989016011993

**Published:** 2016-07-29

**Authors:** Judita Katinaitė, William T. A. Harrison

**Affiliations:** a Department of Chemistry, University of Aberdeen, Meston Walk, Aberdeen AB24 3UE, Scotland

**Keywords:** crystal structure, unsymmetrical di­methyl­hydrazine, protonation pattern, hydrogen bonds

## Abstract

We describe the syntheses and crystal structures of two mol­ecular salts containing the 1,1-di­methyl­hydrazinium cation, namely 1,1-di­methyl­hydrazin-1-ium bromide, C_2_H_9_N_2_^+^·Br^−^, (I), and 2,2-di­methyl­hydrazin-1-ium di­hydrogen phosphite, C_2_H_9_N_2_^+^·H_2_PO_3_^−^, (II). In (I), the cation is protonated at the methyl­ated N atom and N—H⋯Br hydrogen bonds generate [010] chains in the crystal. In (II), the cation is protonated at the terminal N atom and cation-to-anion N—H⋯O and anion-to-anion O—H⋯O hydrogen bonds generate (001) sheets.

## Abstract 2

The crystal structures of two salts of unsymmetrical dimethyl hydrazine show different protonation patterns of the cation.

## Chemical context   

Unsymmetrical di­methyl­hydrazine (1,1-di­methyl­hydrazine; C_2_H_8_N_2_; UDMH) is a colourless liquid at room temperature and pressure with a strong and unpleasant ammonia-like or fishy smell. The best known application of this compound is the fuel (reducing agent) in hypergolic rocket fuels (Edwards, 2003 ▸), where it can be used alone or mixed with hydrazine: the latter formulation (trade name ‘Aerozine 50’) was used by the Apollo lunar modules to begin their homeward journeys from the moon.

Chemically, both nitro­gen atoms in UDMH bear lone pairs of electrons, which can act as weak bases to accept protons and therefore result in the formation of mol­ecular salts when reacted with acids. The first crystal structure of a UDMH salt was reported by Klapötke *et al.* (1999 ▸), who prepared 1,1-dimetylhydrazinium azide as a possible high-energy-density material with military applications; the methyl­ated UDMH nitro­gen atom is protonated and the components are linked by strong N—H⋯N hydrogen bonds in the crystal. However, this salt exhibited pronounced hygroscopic behaviour and had a low melting point of 311 K, which deemed it unsuitable for such uses. The nitrate salt of UDMH, which may be a decomposition product of hypergolic fuels, was prepared soon afterwards by the same workers (De Bonn *et al.*, 2001 ▸) by a low-temperature, non-aqueous synthesis: anhydrous nitric acid and UDMH were separately dissolved in di­chloro­methane at 195 K and the solutions mixed at the same temperature. The resulting hygroscopic salt, 1,1-di­methyl­hydrazinium nitrate, is protonated at the methyl­ated nitro­gen atom and features N—H⋯O hydrogen bonds in its crystal structure.

Merkoulov *et al.* (2005 ▸) synthesized 1,1-di­methyl­hydrazinium chloride by reacting liquid UDMH with HCl dissolved in diethyl ether: its crystal structure consists of two independent cations and two chloride anions in the asymmetric unit. The cation is protonated at the methyl­ated nitro­gen atom and a dense network of strong N—H⋯Cl and weak C—H⋯Cl hydrogen bonds helps to consolidate the packing in the crystal. A salt with a more complicated counter-ion was synthesised by Mu *et al.* (2011 ▸): the addition of liquid UDMH to a solution of picric acid in ethanol at room temperature yielded 1,1-di­methyl­hydrazinium picrate. As before, the UDMH protonates at the methyl­ated nitro­gen atom and cation-to-anion N—H⋯O hydrogen bonds help to establish the packing.
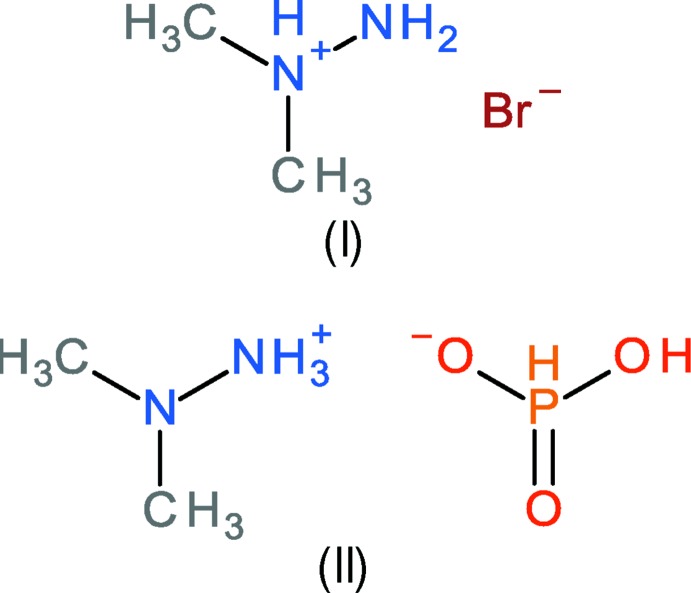


As an extension of these studies, we now describe the syntheses and crystal structures of 1,1-di­methyl­hydrazin-1-ium bromide, C_2_H_9_N_2_^+^·Br^−^ (I) and 2,2-di­methyl­hydrazin-1-ium di­hydrogen phosphite, C_2_H_9_N_2_^+^·H_2_PO_3_^−^ (II).

## Structural commentary   

**Figure 1:**
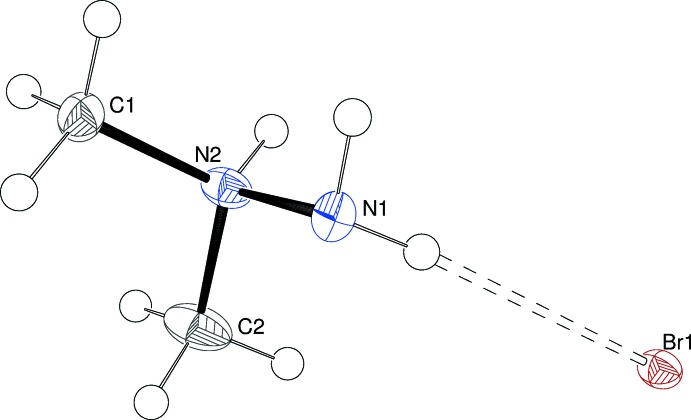
The mol­ecular structure of (I), showing 50% displacement ellipsoids. The N—H⋯Br hydrogen bond is indicated by a double-dashed line (Table 1 ▸).

Compound (I) crystallizes in space group *I*2/*a* (non-standard setting of *C*2/*c*) with one cation and one bromide anion in the asymmetric unit (Fig. 1 ▸). The cation is protonated at the central N2 atom, as seen in previous UDMH salts referred to above. The N1—N2 bond length [1.4478 (19) Å] is slightly shorter than the C—N bond lengths [1.482 (2) and 1.485 (2) Å]. N2 is displaced from N1, C1 and C2 by 0.4834 (16) Å and the C—N—C bond angle [111.38 (14)°] is slightly greater than the C—N—N angles [108.93 (12) and 108.97 (14)°]. The H atoms attached to N1 point away from the carbon atoms [C1—N2—N1—H2*n* = −175.7 (2); C2—N2—N1—H1*n* = 178.0 (2)°] and the N2—H3*n* bond bis­ects the N1H_2_ group [H3*n*—N2—N1—H1*n* = 61 (2)°].

**Figure 2:**
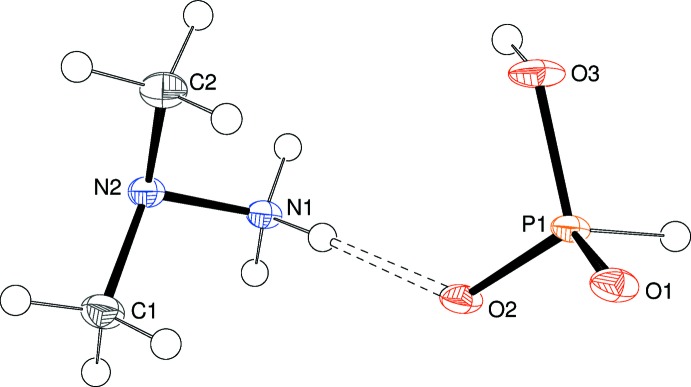
The mol­ecular structure of (II), showing 50% displacement ellipsoids. The N—H⋯O hydrogen bond is indicated by a double-dashed line (Table 2 ▸).

Compound (II) crystallizes in space group *Pna*2_1_ with one cation and one di­hydrogen phosphite anion in the asymmetric unit (Fig. 2 ▸). In this case, the cation is protonated at the terminal N atom rather than the central N atom, which has not been seen previously in UDMH salts. The N1—N2 bond length is 1.454 (3) Å and the C—N bond lengths are 1.462 (3) and 1.463 (3) Å. The geometry about N2 is pyramidal and this atom is displaced from N1, C1 and C2 by 0.504 (2) Å. The bond angles about N2 show the same trend as those in (I): C—N—C = 110.69 (18); C—N—N = 107.62 (17) and 107.94 (18)°. Two of the H atoms attached to N1 have almost the same locations as the corresponding atoms in (I), whereas the third bis­ects the C1—N2—C2 grouping [C1—N2—N1—H3*n* = −62°]. In the anion, the P1—O3 bond length of 1.5638 (16) Å is typical (Harrison, 2003 ▸) for the protonated O atom in a di­hydrogen phosphite group whereas P1—O1 [1.4982 (15) Å] and P1—O2 [1.5003 (16) Å] are almost the same length, indicating the expected delocalization (resonance) of the negative charge over these two O atoms. The O—P—O bond angle for the unprotonated oxygen atoms [116.76 (9)°] is significantly larger than the O—P—OH angles [106.37 (9) and 111.46 (9)°], as seen previously for similar species (Harrison, 2003 ▸). P1 is displaced from its attached O atoms by 0.4510 (13) Å.

## Supra­molecular features   

**Figure 3:**
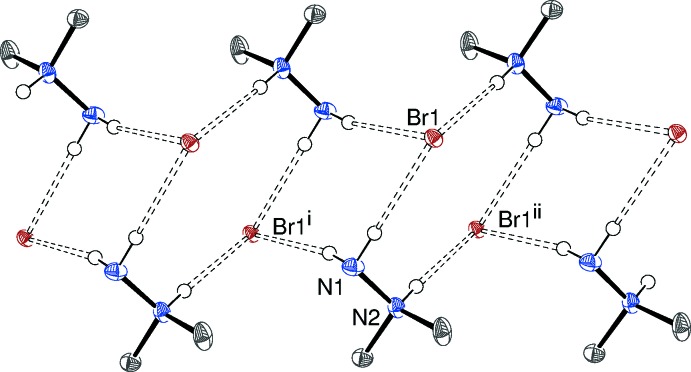
Partial packing diagram for (I), showing the formation of [010] chains linked by N—H⋯Br hydrogen bonds. C-bound H atoms are omitted for clarity. Symmetry codes as in Table 1 ▸.

**Figure 4:**
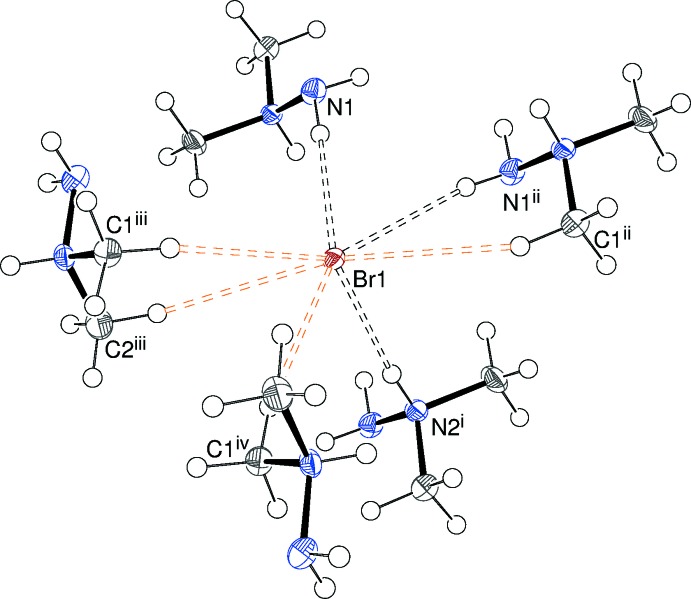
The environment of the bromide ion in the crystal of (I). [Symmetry codes: (i) 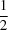 − *x*, 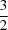 − *y*, 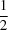 − *z*; (ii) 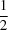 − *x*, 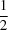 − *y*, 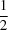 − *z*; (iii) −*x*, 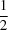 + *y*, 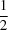 − *y*; (iv) *x*, 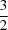 − *y*, *z* − 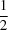.] Note that each of the five cations has a different bonding mode: η^1^ N1, N2 and C1 and η^2^ N1 + C1 and C1 + C2.

In the crystal of (I), N—H⋯Br hydrogen bonds (Table 1 ▸) link the components into [010] chains (Fig. 3 ▸): each Br^−^ ion accepts three N—H⋯Br bonds and alternating, centrosymmetric 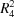
(8) and 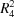
(10) loops occur within the chain. The N2 bond is significantly shorter than the N1 bonds, which may be due to the positive charge residing on N2: this was also observed in the structure of the nitrate salt (de Bonn *et al.*, 2001 ▸). There are also several weak C—H⋯Br contacts (Table 1 ▸) in (I); the weak and strong inter­actions result in each bromide ion accepting a total of seven hydrogen bonds (Fig. 4 ▸).

**Figure 5:**
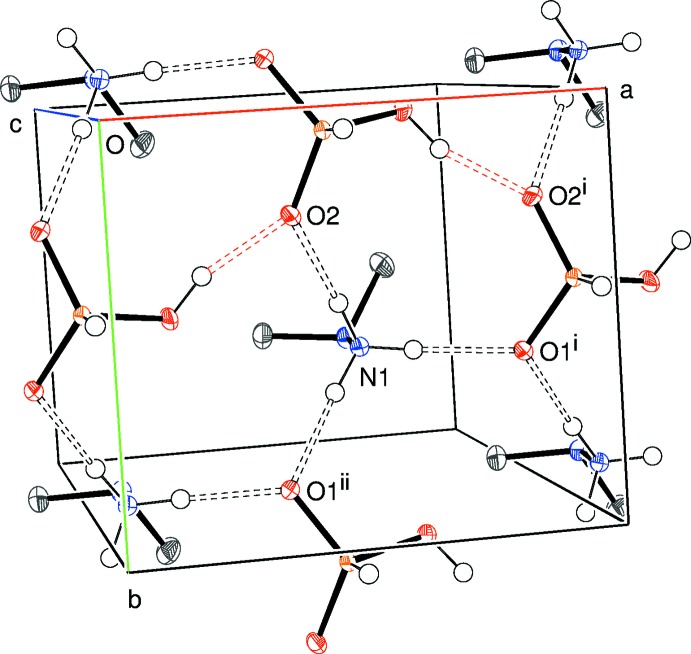
Partial packing diagram for (II), showing part of an (001) sheet. Symmetry codes as in Table 2 ▸.

The crystal structure of (II) appears to correlate with the novel protonation pattern of the C_2_H_9_N_2_^+^ cation: the three H atoms attached to N1 each partake in a strong, near-linear N—H⋯O hydrogen bond to nearby H_2_PO_3_^−^ anions (Table 2 ▸). The anions are linked into [100] chains by O—H⋯O hydrogen bonds with adjacent anions in the chain related by *a*-glide symmetry. Together, these inter­actions generate (001) sheets (Fig. 5 ▸) As usual (Harrison, 2001 ▸), the P—H grouping of the anion does not participate in hydrogen bonds and the H atom points into the inter-layer region.

## Database survey   

A search of the Cambridge Structural Database (CSD; Groom *et al.*, 2016 ▸) revealed the crystal structures of the four UDMH derivatives cited above: refcodes for the azide, nitrate, chloride and picrate salts are CORRUW, IBOLOA, FOHLUK and AZUXID, respectively.

## Synthesis and crystallization   

Caution! UDMH is toxic, potentially carcinogenic and may form explosive mixtures with oxidizing agents: all appropriate safety measures must be put in place when handling this compound.

To prepare (I), aqueous solutions of UDMH (10 ml, 1.0 *M*) and hydro­bromic acid (10 ml, 1.0 *M*) were mixed at room temperature to yield a colourless solution and colourless rods (to ∼1 mm in length) of (I) grew as the solvent evaporated in a watch glass. These crystals are extremely hygroscopic and should be immediately transferred to a desiccator for storage: if left in air, they absorb enough water to completely dissolve within an hour or two.

To prepare (II), aqueous solutions of UDMH (10 ml, 1.0 *M*) and phospho­rus acid (10 ml, 1.0 *M*) were mixed at room temperature to yield a colourless solution and yellowish slabs of (II) grew as the increasingly viscous solvent slowly evaporated over several days in a watch glass. These crystals are hygroscopic and should be stored in a desiccator. IR: 2383 cm^−1^ (P—H stretch).

The IR spectra of UDMH, (I) and (II) are available as supporting information.

## Refinement   

Crystal data, data collection and structure refinement details are summarized in Table 3 ▸. The N-bound H atoms in (I) were located in difference maps and their positions freely refined; those in (II) were relocated to idealized locations and refined as riding atoms. The O-bound H atom in (II) was located in a difference map and refined as riding, in its as-found relative position. The methyl H atoms were geometrically placed (C—H = 0.98 Å): the –CH_3_ groups were allowed to rotate, but not to tip, to best fit the electron density. The constraint *U*_iso_(H) = 1.2*U*_eq_(carrier) or 1.5*U*_eq_(methyl carrier) was applied in all cases.

## Supplementary Material

Crystal structure: contains datablock(s) I, II, global. DOI: 10.1107/S2056989016011993/su5314sup1.cif

Structure factors: contains datablock(s) I. DOI: 10.1107/S2056989016011993/su5314Isup2.hkl

Structure factors: contains datablock(s) II. DOI: 10.1107/S2056989016011993/su5314IIsup3.hkl

Supporting information file. DOI: 10.1107/S2056989016011993/su5314Isup4.cml

Supporting information file. DOI: 10.1107/S2056989016011993/su5314IIsup5.cml

CCDC references: 1495475, 1495474

Additional supporting information:  crystallographic information; 3D view; checkCIF report

## Figures and Tables

**Table 1:** Hydrogen-bond geometry (Å, °) for (I)

*D*—H⋯*A*	*D*—H	H⋯*A*	*D*⋯*A*	*D*—H⋯*A*
N1—H1*n*⋯Br1^i^	0.89 (2)	2.68 (3)	3.5666 (15)	170.7 (18)
N1—H2*n*⋯Br1	0.89 (2)	2.62 (2)	3.5117 (14)	175.0 (19)
N2—H3*n*⋯Br1^ii^	0.87 (2)	2.39 (2)	3.2490 (13)	173.3 (17)
C1—H1*a*⋯Br1^i^	0.98	3.11	3.9690 (18)	148
C1—H1*b*⋯Br1^iii^	0.98	3.09	4.0175 (19)	158
C1—H1*c*⋯Br1^iv^	0.98	2.90	3.8682 (17)	168
C2—H2*c*⋯Br1^iii^	0.98	3.07	3.9843 (18)	156

Symmetry codes: (i) 
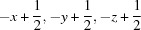
; (ii) 
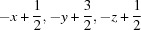
; (iii) 
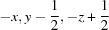
; (iv) 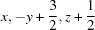.

**Table 2:** Hydrogen-bond geometry (Å, °) for (II)

*D*—H⋯*A*	*D*—H	H⋯*A*	*D*⋯*A*	*D*—H⋯*A*
N1—H1*n*⋯O1^i^	0.91	1.83	2.736 (2)	176
N1—H2*n*⋯O1^ii^	0.91	1.85	2.762 (2)	176
N1—H3*n*⋯O2	0.91	1.91	2.814 (2)	175
O3—H1*o*⋯O2^i^	0.87	1.74	2.568 (2)	159

Symmetry codes: (i) 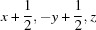; (ii) 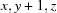.

**Table 3:** Experimental details

	(I)	(II)
Crystal data		
Chemical formula	C_2_H_9_N_2_^+^·Br^−^	C_2_H_9_N_2_^+^·H_2_PO_3_^−^
*M*_r_	141.02	142.10
Crystal system, space group	Monoclinic, *I*2/*a*	Orthorhombic, *P* *n* *a*2_1_
Temperature (K)	100	100
*a*, *b*, *c* (Å)	13.2423 (2), 5.1239 (1), 16.1839 (3)	8.0690 (2), 6.9970 (2), 11.7001 (6)
α, β, γ (°)	90, 94.838 (2), 90	90, 90, 90
*V* (Å^3^)	1094.20 (3)	660.57 (4)
*Z*	8	4
Radiation type	Mo *K*α	Mo *K*α
μ (mm^−1^)	7.36	0.35
Crystal size (mm)	0.23 × 0.09 × 0.09	0.18 × 0.18 × 0.02

Data collection		
Diffractometer	Rigaku Mercury CCD	Rigaku Mercury CCD
Absorption correction	Multi-scan (*CrystalClear*; Rigaku, 2012 ▸)	–
*T*_min_, *T*_max_	0.282, 0.557	–
No. of measured, independent and observed [*I* > 2σ(*I*)] reflections	6485, 1258, 1224	5347, 1395, 1365
*R*_int_	0.029	0.023
(sin θ/λ)_max_ (Å^−1^)	0.649	0.649

Refinement		
*R*[*F* ^2^ > 2σ(*F* ^2^)], *wR*(*F* ^2^), *S*	0.020, 0.051, 1.12	0.025, 0.065, 1.09
No. of reflections	1258	1395
No. of parameters	58	77
No. of restraints	0	1
H-atom treatment	H atoms treated by a mixture of independent and constrained refinement	H-atom parameters constrained
Δρ_max_, Δρ_min_ (e Å^−3^)	0.50, −0.48	0.24, −0.28
Absolute structure	–	Refined as an inversion twin.
Absolute structure parameter	–	0.15 (14)

Computer programs: *CrystalClear* (Rigaku, 2012 ▸), *SHELXS97* (Sheldrick, 2008 ▸), *SHELXL2014* (Sheldrick, 2015 ▸), *ORTEP-3 for Windows* (Farrugia, 2012 ▸) and *publCIF* (Westrip, 2010 ▸).

## supplementary crystallographic information

### (I) 1,1-Dimethylhydrazin-1-ium bromide Crystal data

**Table:**

C_2_H_9_N_2_^+^·Br^−^	*F*(000) = 560
*M**_r_* = 141.02	*D*_x_ = 1.712 Mg m^−^^3^
Monoclinic, *I*2/*a*	Mo *K*α radiation, λ = 0.71073 Å
*a* = 13.2423 (2) Å	Cell parameters from 5743 reflections
*b* = 5.1239 (1) Å	θ = 2.5–27.5°
*c* = 16.1839 (3) Å	µ = 7.36 mm^−^^1^
β = 94.838 (2)°	*T* = 100 K
*V* = 1094.20 (3) Å^3^	Rod, colourless
*Z* = 8	0.23 × 0.09 × 0.09 mm

### (I) 1,1-Dimethylhydrazin-1-ium bromide Data collection

**Table:**

Rigaku Mercury CCD diffractometer	1224 reflections with *I* > 2σ(*I*)
ω scans	*R*_int_ = 0.029
Absorption correction: multi-scan (CrystalClear; Rigaku, 2012)	θ_max_ = 27.5°, θ_min_ = 2.5°
*T*_min_ = 0.282, *T*_max_ = 0.557	*h* = −17→17
6485 measured reflections	*k* = −6→5
1258 independent reflections	*l* = −20→19

### (I) 1,1-Dimethylhydrazin-1-ium bromide Refinement

**Table:**

Refinement on *F*^2^	Secondary atom site location: difference Fourier map
Least-squares matrix: full	Hydrogen site location: mixed
*R*[*F*^2^ > 2σ(*F*^2^)] = 0.020	H atoms treated by a mixture of independent and constrained refinement
*wR*(*F*^2^) = 0.051	*w* = 1/[σ^2^(*F*_o_^2^) + (0.0349*P*)^2^ + 0.3874*P*] where *P* = (*F*_o_^2^ + 2*F*_c_^2^)/3
*S* = 1.12	(Δ/σ)_max_ = 0.001
1258 reflections	Δρ_max_ = 0.50 e Å^−^^3^
58 parameters	Δρ_min_ = −0.48 e Å^−^^3^
0 restraints	Extinction correction: SHELXL2014 (Sheldrick, 2015), Fc^*^=kFc[1+0.001xFc^2^λ^3^/sin(2θ)]^-1/4^
Primary atom site location: structure-invariant direct methods	Extinction coefficient: 0.0151 (6)

### (I) 1,1-Dimethylhydrazin-1-ium bromide Special details

**Table:**

Geometry. All esds (except the esd in the dihedral angle between two l.s. planes) are estimated using the full covariance matrix. The cell esds are taken into account individually in the estimation of esds in distances, angles and torsion angles; correlations between esds in cell parameters are only used when they are defined by crystal symmetry. An approximate (isotropic) treatment of cell esds is used for estimating esds involving l.s. planes.

### (I) 1,1-Dimethylhydrazin-1-ium bromide Fractional atomic coordinates and isotropic or equivalent isotropic displacement parameters (Å^2^)

**Table:**

	*x*	*y*	*z*	*U*_iso_*/*U*_eq_	
N1	0.13506 (11)	0.3029 (3)	0.30499 (9)	0.0197 (3)	
H1n	0.1888 (17)	0.204 (4)	0.3204 (13)	0.024*	
H2n	0.1477 (17)	0.393 (3)	0.2599 (15)	0.024*	
N2	0.13068 (10)	0.4851 (2)	0.37297 (8)	0.0170 (3)	
H3n	0.1867 (17)	0.574 (4)	0.3765 (12)	0.020*	
C1	0.11750 (14)	0.3372 (3)	0.45000 (11)	0.0191 (3)	
H1a	0.1708	0.2047	0.4582	0.029*	
H1b	0.0510	0.2519	0.4454	0.029*	
H1c	0.1220	0.4571	0.4973	0.029*	
C2	0.04544 (15)	0.6693 (3)	0.35325 (15)	0.0278 (4)	
H2a	0.0567	0.7656	0.3025	0.042*	
H2b	0.0416	0.7925	0.3992	0.042*	
H2c	−0.0182	0.5715	0.3450	0.042*	
Br1	0.17057 (2)	0.64136 (3)	0.12100 (2)	0.01501 (11)	

### (I) 1,1-Dimethylhydrazin-1-ium bromide Atomic displacement parameters (Å^2^)

**Table:**

	*U*^11^	*U*^22^	*U*^33^	*U*^12^	*U*^13^	*U*^23^
N1	0.0198 (7)	0.0244 (6)	0.0150 (7)	0.0014 (6)	0.0030 (5)	0.0027 (5)
N2	0.0120 (6)	0.0147 (6)	0.0240 (7)	−0.0019 (5)	−0.0004 (5)	0.0017 (5)
C1	0.0195 (8)	0.0226 (8)	0.0151 (8)	−0.0015 (5)	0.0010 (6)	−0.0008 (5)
C2	0.0175 (9)	0.0173 (8)	0.0479 (12)	0.0030 (6)	−0.0014 (8)	0.0067 (7)
Br1	0.01199 (13)	0.01558 (14)	0.01745 (14)	−0.00069 (4)	0.00116 (7)	0.00263 (4)

### (I) 1,1-Dimethylhydrazin-1-ium bromide Geometric parameters (Å, º)

**Table:**

N1—N2	1.4478 (19)	C1—H1a	0.98
N1—H1n	0.89 (2)	C1—H1b	0.98
N1—H2n	0.89 (2)	C1—H1c	0.98
N2—C1	1.482 (2)	C2—H2a	0.98
N2—C2	1.485 (2)	C2—H2b	0.98
N2—H3n	0.87 (2)	C2—H2c	0.98
			
N2—N1—H1n	103.6 (14)	H1a—C1—H1b	109.5
N2—N1—H2n	108.0 (12)	N2—C1—H1c	109.5
H1n—N1—H2n	108.8 (19)	H1a—C1—H1c	109.5
N1—N2—C1	108.93 (12)	H1b—C1—H1c	109.5
N1—N2—C2	108.97 (14)	N2—C2—H2a	109.5
C1—N2—C2	111.38 (14)	N2—C2—H2b	109.5
N1—N2—H3n	107.4 (13)	H2a—C2—H2b	109.5
C1—N2—H3n	111.9 (13)	N2—C2—H2c	109.5
C2—N2—H3n	108.1 (13)	H2a—C2—H2c	109.5
N2—C1—H1a	109.5	H2b—C2—H2c	109.5
N2—C1—H1b	109.5		

### (I) 1,1-Dimethylhydrazin-1-ium bromide Hydrogen-bond geometry (Å, º)

**Table:**

*D*—H···*A*	*D*—H	H···*A*	*D*···*A*	*D*—H···*A*
N1—H1*n*···Br1^i^	0.89 (2)	2.68 (3)	3.5666 (15)	170.7 (18)
N1—H2*n*···Br1	0.89 (2)	2.62 (2)	3.5117 (14)	175.0 (19)
N2—H3*n*···Br1^ii^	0.87 (2)	2.39 (2)	3.2490 (13)	173.3 (17)
C1—H1*a*···Br1^i^	0.98	3.11	3.9690 (18)	148
C1—H1*b*···Br1^iii^	0.98	3.09	4.0175 (19)	158
C1—H1*c*···Br1^iv^	0.98	2.90	3.8682 (17)	168
C2—H2*c*···Br1^iii^	0.98	3.07	3.9843 (18)	156

Symmetry codes: (i) −*x*+1/2, −*y*+1/2, −*z*+1/2; (ii) −*x*+1/2, −*y*+3/2, −*z*+1/2; (iii) −*x*, *y*−1/2, −*z*+1/2; (iv) *x*, −*y*+3/2, *z*+1/2.

### (II) 2,2-Dimethylhydrazin-1-ium dihydrogen phosphite Crystal data

**Table:**

C_2_H_9_N_2_^+^·H_2_PO_3_^−^	*D*_x_ = 1.429 Mg m^−^^3^
*M**_r_* = 142.10	Mo *K*α radiation, λ = 0.71073 Å
Orthorhombic, *P**n**a*2_1_	Cell parameters from 4031 reflections
*a* = 8.0690 (2) Å	θ = 3.4–27.5°
*b* = 6.9970 (2) Å	µ = 0.35 mm^−^^1^
*c* = 11.7001 (6) Å	*T* = 100 K
*V* = 660.57 (4) Å^3^	Plate, yellow
*Z* = 4	0.18 × 0.18 × 0.02 mm
*F*(000) = 304	

### (II) 2,2-Dimethylhydrazin-1-ium dihydrogen phosphite Data collection

**Table:**

Rigaku Mercury CCD diffractometer	*R*_int_ = 0.023
ω scans	θ_max_ = 27.5°, θ_min_ = 3.4°
5347 measured reflections	*h* = −8→10
1395 independent reflections	*k* = −8→9
1365 reflections with *I* > 2σ(*I*)	*l* = −15→13

### (II) 2,2-Dimethylhydrazin-1-ium dihydrogen phosphite Refinement

**Table:**

Refinement on *F*^2^	Secondary atom site location: difference Fourier map
Least-squares matrix: full	Hydrogen site location: mixed
*R*[*F*^2^ > 2σ(*F*^2^)] = 0.025	H-atom parameters constrained
*wR*(*F*^2^) = 0.065	*w* = 1/[σ^2^(*F*_o_^2^) + (0.0374*P*)^2^ + 0.203*P*] where *P* = (*F*_o_^2^ + 2*F*_c_^2^)/3
*S* = 1.09	(Δ/σ)_max_ < 0.001
1395 reflections	Δρ_max_ = 0.24 e Å^−^^3^
77 parameters	Δρ_min_ = −0.28 e Å^−^^3^
1 restraint	Absolute structure: Refined as an inversion twin.
Primary atom site location: structure-invariant direct methods	Absolute structure parameter: 0.15 (14)

### (II) 2,2-Dimethylhydrazin-1-ium dihydrogen phosphite Special details

**Table:**

Geometry. All esds (except the esd in the dihedral angle between two l.s. planes) are estimated using the full covariance matrix. The cell esds are taken into account individually in the estimation of esds in distances, angles and torsion angles; correlations between esds in cell parameters are only used when they are defined by crystal symmetry. An approximate (isotropic) treatment of cell esds is used for estimating esds involving l.s. planes.
Refinement. Refined as a 2-component inversion twin.

### (II) 2,2-Dimethylhydrazin-1-ium dihydrogen phosphite Fractional atomic coordinates and isotropic or equivalent isotropic displacement parameters (Å^2^)

**Table:**

	*x*	*y*	*z*	*U*_iso_*/*U*_eq_	
N1	0.5416 (2)	0.5852 (2)	0.22552 (17)	0.0132 (4)	
H1n	0.6455	0.5972	0.1962	0.016*	
H2n	0.4779	0.6848	0.2016	0.016*	
H3n	0.4960	0.4735	0.2011	0.016*	
N2	0.5499 (2)	0.5853 (3)	0.34964 (17)	0.0145 (4)	
C1	0.3807 (3)	0.5724 (3)	0.3936 (3)	0.0189 (5)	
H1a	0.3169	0.6834	0.3677	0.028*	
H1b	0.3832	0.5699	0.4773	0.028*	
H1c	0.3286	0.4551	0.3652	0.028*	
C2	0.6471 (3)	0.4186 (3)	0.3845 (2)	0.0206 (5)	
H2a	0.7575	0.4255	0.3499	0.031*	
H2b	0.5912	0.3018	0.3590	0.031*	
H2c	0.6576	0.4169	0.4679	0.031*	
P1	0.45797 (6)	0.05934 (7)	0.10615 (6)	0.01209 (15)	
H1	0.4666	0.0529	−0.0064	0.015*	
O1	0.35870 (18)	−0.1101 (2)	0.14409 (14)	0.0153 (3)	
O2	0.39202 (17)	0.2526 (2)	0.13797 (14)	0.0165 (4)	
O3	0.63746 (19)	0.0285 (2)	0.15307 (17)	0.0196 (4)	
H1o	0.7059	0.1224	0.1417	0.024*	

### (II) 2,2-Dimethylhydrazin-1-ium dihydrogen phosphite Atomic displacement parameters (Å^2^)

**Table:**

	*U*^11^	*U*^22^	*U*^33^	*U*^12^	*U*^13^	*U*^23^
N1	0.0099 (8)	0.0108 (8)	0.0189 (10)	0.0002 (6)	−0.0001 (7)	−0.0003 (7)
N2	0.0114 (9)	0.0142 (9)	0.0180 (11)	0.0005 (6)	−0.0007 (7)	−0.0007 (8)
C1	0.0124 (10)	0.0206 (11)	0.0236 (13)	−0.0007 (8)	0.0021 (10)	−0.0016 (9)
C2	0.0172 (11)	0.0209 (12)	0.0237 (13)	0.0050 (8)	−0.0028 (10)	0.0023 (10)
P1	0.0074 (2)	0.0095 (2)	0.0194 (3)	0.00031 (18)	0.0003 (3)	0.0004 (2)
O1	0.0097 (6)	0.0100 (7)	0.0263 (9)	−0.0005 (6)	0.0011 (6)	0.0013 (6)
O2	0.0097 (6)	0.0112 (7)	0.0286 (10)	0.0017 (6)	−0.0011 (6)	−0.0023 (6)
O3	0.0075 (6)	0.0127 (7)	0.0386 (10)	−0.0009 (6)	−0.0030 (7)	0.0040 (7)

### (II) 2,2-Dimethylhydrazin-1-ium dihydrogen phosphite Geometric parameters (Å, º)

**Table:**

N1—N2	1.454 (3)	C2—H2a	0.98
N1—H1n	0.91	C2—H2b	0.98
N1—H2n	0.91	C2—H2c	0.98
N1—H3n	0.91	P1—O1	1.4982 (15)
N2—C1	1.462 (3)	P1—O2	1.5003 (16)
N2—C2	1.463 (3)	P1—O3	1.5638 (16)
C1—H1a	0.98	P1—H1	1.32
C1—H1b	0.98	O3—H1o	0.8689
C1—H1c	0.98		
			
N2—N1—H1n	109.5	H1b—C1—H1c	109.5
N2—N1—H2n	109.5	N2—C2—H2a	109.5
H1n—N1—H2n	109.5	N2—C2—H2b	109.5
N2—N1—H3n	109.5	H2a—C2—H2b	109.5
H1n—N1—H3n	109.5	N2—C2—H2c	109.5
H2n—N1—H3n	109.5	H2a—C2—H2c	109.5
N1—N2—C1	107.94 (18)	H2b—C2—H2c	109.5
N1—N2—C2	107.62 (17)	O1—P1—O2	116.76 (9)
C1—N2—C2	110.69 (18)	O1—P1—O3	106.37 (9)
N2—C1—H1a	109.5	O2—P1—O3	111.46 (9)
N2—C1—H1b	109.5	O1—P1—H1	107.3
H1a—C1—H1b	109.5	O2—P1—H1	107.3
N2—C1—H1c	109.5	O3—P1—H1	107.3
H1a—C1—H1c	109.5	P1—O3—H1o	115.5

### (II) 2,2-Dimethylhydrazin-1-ium dihydrogen phosphite Hydrogen-bond geometry (Å, º)

**Table:**

*D*—H···*A*	*D*—H	H···*A*	*D*···*A*	*D*—H···*A*
N1—H1*n*···O1^i^	0.91	1.83	2.736 (2)	176
N1—H2*n*···O1^ii^	0.91	1.85	2.762 (2)	176
N1—H3*n*···O2	0.91	1.91	2.814 (2)	175
O3—H1*o*···O2^i^	0.87	1.74	2.568 (2)	159

Symmetry codes: (i) *x*+1/2, −*y*+1/2, *z*; (ii) *x*, *y*+1, *z*.
